# Cannabis Use and Fungal Infections in a Commercially Insured Population, United States, 2016

**DOI:** 10.3201/eid2606.191570

**Published:** 2020-06

**Authors:** Kaitlin Benedict, George R. Thompson, Brendan R. Jackson

**Affiliations:** Centers for Disease Control and Prevention, Atlanta, Georgia, USA (K. Benedict, B.R. Jackson);; University of California Davis Medical Center, Davis, California, USA (G.R. Thompson III)

**Keywords:** cannabis, cannabis use, aspergillosis, blastomycosis, coccidioidomycosis, cryptococcosis, histoplasmosis, mucormycosis, fungi, fungal infections, health insurance, commercially insured population, United States

## Abstract

Case reports have identified invasive fungal diseases in persons who use cannabis, and fungal contamination of cannabis has been described. In a large health insurance claims database, persons who used cannabis were 3.5 (95% CI 2.6–4.8) times more likely than persons who did not use cannabis to have a fungal infection in 2016.

Cannabis can contain fungal pathogens that cause serious and often fatal infections in persons with immunocompromising conditions, such as cancer, transplant, or infection with HIV (*1*). In these patients, some reasons for using cannabis include pain and nausea relief and appetite stimulation. The frequency of fungal infections associated with cannabis is unknown but is a growing concern as more states legalize its medicinal and recreational use. We used health insurance claims data from 2016 to evaluate the prevalence of fungal infection diagnosis codes among persons who use cannabis and persons who do not use cannabis and to compare demographic and clinical features between these 2 groups.

## The Study

The 2016 IBM MarketScan Research Databases (https://www.ibm.com/products/marketscan-research-databases) include claims from outpatient visits and prescriptions and hospitalizations for >27 million employees, dependents, and retirees throughout the United States. MarketScan represents one of the largest collections of such data in the country and captures patient interactions across the full spectrum of healthcare. We used Treatment Pathways, a web-based platform (https://www.ibm.com/us-en/marketplace/marketscan-treatment-pathways), which enable users to query data for persons whose health insurance plans or employers contribute prescription drug data to MarketScan. Because data are fully deidentified, this analysis was not subject to review by the Centers for Disease Control and Prevention institutional review board.

We studied persons with continuous insurance enrollment in 2016, excluding those with diagnosis codes from the International Classification of Diseases, 10th Revision, Clinical Modification (ICD-10-CM), for presumed ocular histoplasmosis syndrome (H32 plus B39.4 or B39.9) assigned at an eye care provider (*2*). We identified patients with ICD-10-CM codes for mold infections (aspergillosis [B44], mucormycosis [B46]) and certain other fungal infections (blastomycosis [B40], coccidioidomycosis [B38], cryptococcosis [B45], histoplasmosis [B39]) among persons who used cannabis (F12.1, F12.2, F12.9) and persons who did not use cannabis. We further explored differences between ICD-10-CM codes for cannabis abuse or dependence (F12.1 and F12.2) and unspecified cannabis use (i.e., without mention of abuse or dependence) (F12.9). We defined immunocompromised status as HIV (B20, O9872, O9873), solid organ or hematopoietic stem cell transplant (Z94, T86), malignant neoplasms (C00–C80 excluding C44), and hematologic malignancies (C81–C96) and also identified tobacco use (Z27.0 or F17.2). We analyzed categorical variables by using χ^2^ tests and logistic regression.

Forty (0.08%) of 53,217 persons who used cannabis and 6,294 (0.03%) of 21,559,558 persons who did not use cannabis had a fungal infection (odds ratio [OR] 2.6, 95% CI 1.9–3.5). After adjusting for age and immunocompromised status, the adjusted OR (aOR) was 3.5 (95% CI 2.6–4.8). Specifically, persons who use cannabis were more likely than persons who did not use cannabis to have mold infections (0.03% vs. 0.01%; OR 3.4, 95% CI 2.1–5.3, aOR 4.6, 95% CI 2.9–7.4) and other fungal infections (0.04% vs. 0.02%; OR 2.2, 95% CI 1.4–3.3, aOR 2.9, 95% CI 1.9–4.5).

Among patients with fungal infections, persons who used cannabis were significantly younger than persons who did not use cannabis (median age 41.5 years vs. 56.0 years; p<0.001), more likely to be immunocompromised (43% vs. 21%; p<0.001), more likely to be hospitalized on the fungal infection diagnosis date (40% vs. 13%; p<0.001), and more likely to have tobacco use codes (40% vs. 9%; p<0.001) (Table). Sixty percent (n = 24) of persons who used cannabis and had fungal infections had cannabis abuse or dependence codes, compared with 79% of persons who used cannabis and did not have fungal infections, and 48% (n = 19) of persons who used cannabis and had fungal infections had unspecified cannabis use codes, compared with 29% of persons who used cannabis and did not have fungal infections. Persons who used cannabis and had fungal infections and unspecified cannabis use codes were older (median age 52 years vs. 28 years) and more frequently immunocompromised (63% vs. 25%) than persons who used cannabis and had dependence codes.

**Table:** Characteristics of patients with fungal infections, by cannabis use status, United States, 2016*

Characteristic	Persons who use cannabis, n = 40	Persons who do not use cannabis, n = 6,294	p value
Median age, y (range)	41.5 (7–70)	56 (0–99)	<0.001
0–17	1 (3)	341 (5)	
18–34	16 (40)	659 (10)	
35–44	4 (10)	745 (12)	
45–54	5 (13)	1,226 (19)	
55–64	13 (33)	1,816 (29)	
>65	1 (3)	1,507 (24)	
Sex			
M	25 (63)	3,078 (49)	0.086
F	15 (37)	3,216 (51)	
US Census Region†			0.964
Northeast	5 (13)	689 (11)	
Midwest	10 (25)	1,581 (25)	
South	12 (30)	2,099 (33)	
West	13 (33)	1,915 (30)	
Immunocompromised	17 (43)	1,303 (21)	<0.001
Inpatient on fungal infection diagnosis date	16 (40)	820 (13)	<0.001
Type of fungal infection			
Aspergillosis	17 (43)	2,091 (33)	
Blastomycosis	1 (3)	218 (3)	
Coccidioidomycosis	10 (25)	1,661 (26)	
Cryptococcosis	4 (10)	338 (5)	
Histoplasmosis	7 (18)	1,945 (31)	
Mucormycosis	1 (3)	82 (1)	
Tobacco use	16 (40)	558 (9)	<0.001

*Values are no. (%) unless otherwise indicated. †Of primary beneficiary’s residence.

## Conclusions

In this large commercially insured population in the United States, cannabis use was associated with a higher prevalence of certain fungal infections. Although these infections were uncommon, they can result in substantial illness and even death, particularly in immunocompromised persons.

Several hypotheses could explain our findings. First, on the basis of immunocompromised status and hospitalizations, persons who used cannabis appeared to be sicker than persons who did not use cannabis and were therefore presumably at higher risk for fungal infections in general. Some persons who used cannabis might be using medical cannabis to help manage their underlying conditions. In this analysis, it was not possible to determine the source of infection, although contaminated cannabis has been previously implicated in aspergillosis, mucormycosis, and cryptococcal meningitis (*3*–*5*). We are not aware of any reports of blastomycosis, histoplasmosis, or coccidioidomycosis acquired from contaminated cannabis. However, a small risk likely exists; 1 histoplasmosis outbreak occurred in a cannabis field (*6*), and fomites, such as hay and vegetables, are involved in rare coccidioidomycosis cases (*7*). Another possible explanation is that smoking-induced structural and immunological lung damage confers increased susceptibility to infection (*8*), although the lung effects of cannabis might differ from those of tobacco (*9*). Confounding by tobacco smoking might be another explanation because tobacco use is typically more common among persons who use cannabis (*10*). Tobacco can also be contaminated with fungi, possibly to a lesser extent than cannabis (*11*).

Our results could also reflect medical coding artifacts. In general, cannabis use is likely greatly underrepresented by ICD codes (*12*), supported by the finding that <0.3% of our study population had cannabis use codes, whereas ≈9% of the US population reported using cannabis in the past month (*13*). Although ICD-10-CM codes cannot distinguish between medical and recreational cannabis use, the higher frequency of immunocompromising conditions and older age among persons who use cannabis and persons who used cannabis and had unspecified cannabis use codes suggests medical cannabis use among some of these patients. We were also unable to differentiate smoking cannabis from other modes of use (e.g., ingestion), which is relevant because smoking might lead to greater fungal exposure through inhalation. Injection drug use, which also might be more common among persons who use cannabis, is an emerging risk factor for some fungal infections, such as invasive candidiasis, although this mode of acquisition seems less likely for the fungal infections described here, which are typically acquired through inhalation. Another limitation is that we did not evaluate immunocompromised status associated with medications such as corticosteroids and tumor necrosis factor inhibitors.

The similar geographic distribution of fungal infections between persons who use cannabis and persons who did not use cannabis is notable because state laws vary substantially regarding medical and recreational use. There is more legalization overall in the western and northeastern United States.

Despite the limitations inherent in administrative data and our inability to infer causality between cannabis use and fungal infections, our study adds to emerging evidence about this association. This finding is consistent with a recommendation that solid organ transplant recipients avoid smoking cannabis (*14*). Patients with other immunocompromising conditions should be also aware of the possible link between cannabis smoking and fungal infections and might also consider avoiding this exposure. Physicians should remain aware of the possible link between fungal infections and cannabis use.
